# Expression of Flotilin-2 and Acrosome Biogenesis Are Regulated by MiR-124 during Spermatogenesis

**DOI:** 10.1371/journal.pone.0136671

**Published:** 2015-08-27

**Authors:** Yibo Wu, Ahong Zhong, Haoyu Zheng, Min Jiang, Zhengrong Xia, Jinjin Yu, Ling Chen, Xiaoyan Huang

**Affiliations:** 1 Department of Reproductive Medicine, Affiliated hospital of Jiangnan University, Wuxi, Jiangsu Province, China; 2 Department of Obstetrics and Gynecology, The Fourth Affiliated Hospital of Soochow University, Wuxi, Jiangsu Province, China; 3 State Key laboratory of Reproductive Medicine, Department of Histology and Embryology, Nanjing Medical University, Nanjing, Jiangsu Province, China; Clermont-Ferrand Univ., FRANCE

## Abstract

MicroRNAs (miRNAs) are a class of short non-coding RNA molecules, which diversely regulate gene expression in organisms. Although the regulatory role of these small RNA molecules has been recently explored in animal spermatogenesis, the role of miR-124 in male germ cells is poorly defined. In our previous study, flotillin-2 was investigated as a novel Golgi-related protein involved in sperm acrosome biogenesis. The current study was designed to analyze the contribution of miR-124 in the regulation of flotillin-2 expression during mouse acrosome biogenesis. Luciferase assays revealed the target effects of miR-124 on flotillin-2 expression. Following intratesticular injection of miR-124 in 3-week-old male mice, quantitative real-time RT-PCR and western blot analysis were employed to confirm the function of miR-124 in regulating flotillin-2 after 48 hours. Sperm abnormalities were assessed 3 weeks later by ordinary optical microscopy, the acrosome abnormalities were also assessed by PNA staining and transmission electron microscopy. The results showed the proportion of sperm acrosome abnormalities was significantly higher than that of the control group. The expression of flotillin-2 and caveolin-1 was significantly downregulated during acrosome biogenesis. These results indicated that miR-124 could potentially play a role in caveolin-independent vesicle trafficking and modulation of flotillin-2 expression in mouse acrosome biogenesis.

## Introduction

Spermatogenesis is the process of the development of sperm from undifferentiated germ cells through a precisely-regulated, well-coordinated mechanism[1,2]. It involves three phases: mitosis, meiosis and spermiogenesis. Spermiogenesis can be divided into four phases referred to as the Golgi, capping, acrosomal and maturation stages[3,4]. Biogenesis of the acrosome, a sperm-specific organelle essential for fertilization and development initiation[5], is a remarkable biological event during spermiogenesis. Acrosome biogenesis covers the transport and fusion of Golgi-derived proacrosomal vesicles to form an acrosome sac which is tightly bound to the nuclear envelope. It is known that the vesicular transportation plays an important role during acrosome biogenesis[6,7]. In our previous study, the localization and role of flotillin-2 in spermiogenesis was investigated, and flotillin-2 was found to be a novel Golgi-related protein involved in sperm acrosome biogenesis[8].

Caveolin-1 is a small integral membrane protein that has been implicated in numerous functions including cell signaling, lipid metabolism and vesicular transport[9]. It has been detected in mice sperm and considered to be involve in acrosome biogenesis[10]. Recent research has suggested that flotillin-2 regulates the expression of caveolin-1 in lung cancer[11]. However, the role of caveolin-1 during acrosome biogenesis is unknown.

MiRNAs are a class of approximately 21-nucleotide-long, non-coding, single-stranded RNA molecules that inhibit the translation of mRNAs by specifically binding to complementary sequences[12], which were originally discovered more than 20 years ago in the *nematode Caenorhabditis elegans*[13]. During the past decade, miRNAs have been found to perform a pivotal role in various biological processes, including cell proliferation[14], differentiation[15,16], and apoptosis[17]. Growing interest in the regulatory role of these small RNA molecules is being driven by recent evidence from several studies, which showed the importance of microRNAs in the regulation of spermatogenesis. It has been reported that miR-146, miR-221, miR-222 and miR-383 regulate gene expression during this process[18–20]. Although miR-124 was identified 12 years ago, its biological function has been recently investigated. MiR-124, a brain-enriched miRNA, was first found to be involved in stem cell regulation and neurodevelopment[21,22]. It regulates early neurogenesis in the optic vesicle and forebrain[23], which is involved in synaptic and cargo vesicle transport in vertebrates in squid lenses; and it was also detected in the regulation of the developing mouse ovarian follicle[24,25], yet its role in male germ cells is poorly described.

According to the information above, we aimed to assess the hypothesis that miR-124 could downregulate the expression of flotillin-2 via targeted binding to its 3’UTR region, and could therefore affect the vesicular transport via regulation of caveolin-1 expression, ultimately leading to acrosome abnormalities.

This study was designed to analyze the contribution of miR-124 in the regulation of flotillin-2 expression during acrosome biogenesis in mouse testes. Bioinformatics analysis predicted that miR-124 potentially regulates the expression of flotillin-2. The effect of miR-124 on flotillin-2 expression was investigated in vitro using a dual luciferase reporter assay system and in vivo using intratesticular injection into 3-week-old male mice. The expression of flotillin-2 and caveolin-1 was significantly downregulated. A number of sperm in mice treated with a miR-124 mimic had abnormal acrosome morphologies. This study provides insights into a novel molecular mechanism of miR-124, with an influence on flotillin-2 expression and involvement in caveolin-independent vesicle trafficking during mouse acrosome biogenesis.

## Materials and Methods

This study was performed in strict accordance with the recommendations in the Guide for the Care and Use of Laboratory Animal of Nanjing Medical University. The protocol was approved by the Institutional Animal Care and Use Committee of Nanjing Medical University (Permit Number: IACUC-1401001). All surgery was performed under sodium pentobarbital anesthesia, and all efforts were made to minimize suffering.

### Animals

Twenty male ICR 3-week-old mice were obtained from the Animal Center of Nanjing Medical University. Mice were housed under pathogen-free conditions with unlimited access to food and water.

### Bioinformatics analysis

Analysis of miR-124 predicted targets was determined using the algorithms of TargetScan 5.1 (http://www.targetscan.org/). According to the algorithms, the flotillin-2 gene was predicted to be a potential direct target of miR-124.

### Plasmid construction

For the construction of the reporter vector, the 3ʹ-UTR of mouse flotillin-2 was amplified and the PCR fragment was digested with XhoI and BamHI. The construct containing site mutations was made by Quick Change site-directed mutagenesis. The constructs used were verified by sequencing. Primer sets and target sequence are shown in S1 Fig.

### Cell culture

The immortalized mouse spermatocyte cell line (GC2-spd; CRL-2196) was purchased from American Type Culture Collection (ATCC; Manassas, VA, USA) and cultured in 10% Dulbecco's modified Eagle’s medium (DMEM; Gibco BRL, Grand Island, NY, USA), 10% fetal bovine serum (FBS) and 1% penicillin-streptomycin and was passaged every 3 days. Cells were grown at 37°C in a 5% CO2 atmosphere.

### Luciferase assays

Cells were trypsinized and plated into 96-well plates at a density of 2×10^4^(100μL) cells/well in antibiotic-free medium. The following day, cells were transfected with either miR-124 mimics or a non-specific miRNA negative control (sequences shown in S2 Fig) according to the manufacturer’s protocol. Twenty-four hours after transfection, firefly and Renilla luciferase activities were measured using a luciferase reporter assay system (E1910, Promega, USA).

### RNA extraction and reverse transcriptase PCR (RT-PCR)

Total RNA was extracted from freshly isolated lysates of mouse testis and brain using Trizol (Invitrogen, Carlsbad, CA, USA) according to the manufacture’s recommendations. For miRNA analysis, total RNA was reverse transcribed into cDNA using a specific stem-loop real-time PCR miRNA kit (Ribobio, Guangzhou, China). RT- PCR was performed using the Platinum SYBR Green qPCR SuperMix-UDG system (Invitrogen, CA, USA) on an Applied Biosystems 7900 system. The miR-124 primers are shown below. PCR products were separated by electrophoresis on 2% agarose gels.

### Intratesticular injection of miRNA

Intratesticular injection was performed as previously described[23,24]. 3-week-old male mice were anesthetized with sodium pentobarbital and the testes were then exteriorized through an approximately 5 mm midline abdominal incision. Approximately 3–5 μL of 0.1 pM miR-124 mimic or a negative control miRNA (Shenzhen Huaanpingkang Biotechnology Company, Shenzhen, China) mixed with indicator (0.4% trypan blue) was injected into the seminiferous tubules via rete testis injection. Following injection, the testes were replaced in the abdomen, the incisions were sutured and the mice were allowed to recover and housed.

Forty-eight hours after the injection of miRNA, flotillin-2 protein expression was quantified by western blot analysis of the seminiferous tubule lysate. In addition, miR-124 expression of the seminiferous tubule lysate was analyzed using qRT-PCR to verify the efficiency of miRNA in vivo. Three weeks later, epididymal sperm morphology was classified after euthanasia.

### Quantitative real-time PCR (qRT-PCR)

Total RNA was isolated from samples using Trizol (Invitrogen, USA). The yield and quality of RNA samples were determined using the NanoDrop 2000 Spectrophotometer (Thermo Scientific, USA). For microRNA analysis, total RNA was reverse transcribed into cDNA using a specific stem-loop real-time PCR miRNA kit (Ribobio, Guangzhou, China). qRT-PCR was performed using the Platinum SYBR Green qPCR SuperMix-UDG system (Invitrogen, USA) on an Applied Biosystems 7900 Real-Time PCR system. 5S rRNA was used as an internal control and all samples were normalized to internal controls. Quantification of the fold change in gene expression was determined by the relative quantification method. Primers of miR-124 and 5S rRNA used in this study are listed as follows:

miR-124 stem-loop RT: 5’-GTCGTATCCAGTGCGTGTCGTGGAGTCGGCAATTGCACTGGATACGACGGCATTC -3’;

miR-124 forward: 5’-GGTAAGGCACGCGGT-3’;

miR-124 reverse: 5’-CAGTGCGTGTCGTGGAGT-3’;

5S rRNA stem-loop RT: 5’-GTCGTATCCAGTGCAGGGTCCGAGGTATTCGCACTGGATACGACCAGGCG-3’;

5S rRNA forward: 5’-CTGGTTAGTACTTGGACGGGAGAC-3’;

5S rRNA reverse: 5’-GTGCAGGGTCCGAGGT-3’

Flotillin-2 forward: 5’- GGAGGCTGTTGTGGTTCTGA-3’

Flotillin-2 reverse: 5’- GTCTCTACGTCCTCACAGCG-3’

β-actin forward: 5’- CCGTAAAGACCTCTATGCC-3’

β-actin reverse: 5’- CTCAGTAACAGTCCGCCTA-3’

### Western blot analysis

The testes of male ICR mice were solubilized in lysis buffer (7 mol/L urea, 2 mol/L thiourea, 4% (W/V) CHAPS and 2% (W/V) DTT), in the presence of 1% (W/V) protease inhibitor cocktail (Pierce Biotechnology, Rockford, IL,USA), and were then homogenized and sonicated. The mixture was placed on a shaker at 4°C for 1 h, and insoluble matter was removed by centrifugation at 40,000 g for 1 h at 4°C. The protein concentration of each sample was quantified by the Bradford protein assay using bovine serum albumin as a standard.

Samples containing 100 μg protein were electrophoresed on 4–20% polyacrylamide gradient gels, followed by transfer onto polyvinylidene difluoride membranes (GE Healthcare, San Francisco, CA, USA), blocked in triethanolamine buffered saline (TBS) containing 5% non-fat milk for 2 hours. Membranes were incubated overnight at 4°C with 1:1000 anti-flotillin-2 (rabbit polyclonal, ab135389, Abcam, Hongkong, China), 1:500 anti-flotillin-1 (rabbit monoclonal, ab133497, Abcam, Hongkong, China), 1:500 anti-caveolin-1 (mouse monoclonal, SAB4200216, Sigma Aldrich, USA) and 1:2000 anti-GAPDH (mouse monoclonal, KC-5g5, Shanghai Kangchen Biotechnology Company, Shanghai, China) in TBS containing 5% non-fat milk powder. Expression of GAPDH was used as a loading control. The following day, blots were washed and incubated with horseradish peroxidase (HRP)-conjugated goat anti-rabbit IgG (Beijing Zhongshan Biotechnology Company, Beijing, China) and the protein bands were detected using the enhanced chemiluminescence (ECL) Western Blot Detection Kit and AlphaImager (GE Healthcare, San Francisco, CA, USA).

### Epididymal sperm morphology classification

Three weeks after injection of miRNAs, spermatozoa were isolated from the cauda epididymis, then spread on slides, fixed in 4% paraformaldehyde, and stained with hematoxylin and eosin at room temperature before morphological observation. Deformities were classified as previously described[26,27]. Six hundred sperm were evaluated in both groups respectively. Differences between the groups were evaluated using Student's t test. P values less than 0.05 were considered significant.

For immunofluorescence detection of the acrosome, sperm suspensions (20μl) were spread onto Superfrost Plus slides, air-dried, and fixed with 4% paraformaldehyde in PBS. The sections were then washed three times (5min/wash) in PBS, exposed to blocking buffer (5% BSA in PBS) for 1 h at RT, and incubated with PNA-FITC [1:50; L7381, Sigma Aldrich, USA] for 30min. After PBS washes for three times (5min/wash), slides were counterstained with Hoechst to visualize nuclei. Fluorescence microscopic imaging was conducted using a fluorescence microscope (LSM700, Carl Zeiss). Three hundred sperm were evaluated in both groups respectively. Differences between the groups were evaluated using Student's t test. P values less than 0.05 were considered significant.

Transmission electron microscopy was performed for ultra-structural examination of epididymal sperm. Spermatozoa from the cauda epididymis were fixed in 5% glutaraldehyde, postfixed with 2% osmium tetroxide, and embedded in Araldite. Ultrathin sections were cut and stained with uranyl acetate and lead citrate. Samples were analyzed with an electron microscope (JEM.1010; JEOL, Tokyo, Japan).

## Results

### MiR-124 downregulated flotillin-2 expression through directly targeting its 3ʹ-UTR

**Fig 1:**
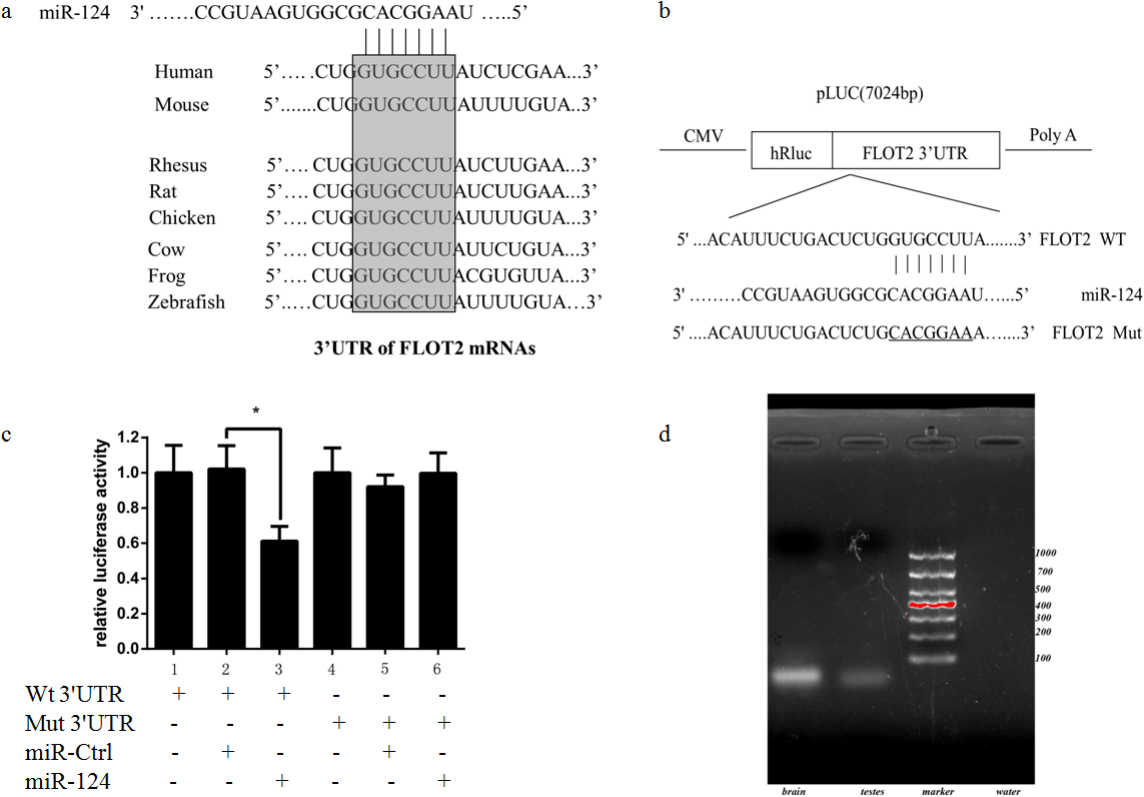
MiR-124 downregulates flotillin-2 expression by directly targeting its 3ʹ-UTR in mouse testicular tissue. (a) Putative duplex formation between miR-214 and the flotillin-2 3ʹ-UTR. The binding site is conserved between several mammalian species (in bold and boxed). (b) Assay constructs, wild-type and mutated target sequences of flotillin-2. (c) MiR-124 downregulated the luciferase activity of the flotillin-2 3ʹ-UTR construct, whereas luciferase activity was not significantly decreased in the target region of the mutated mut 3ʹ-UTR construct *p < 0.05 compared with control (two-tailed). Error bars indicate SD. (d) MiR-124 expression in mouse brain tissue and mouse testicular tissue.

In order to illustrate the molecular mechanism responsible for flotillin-2 regulation, an online tool named TargetScan was used to search for putative miRNAs targeted to flotillin-2. MiR-124 was selected as one of the candidate regulators of flotillin-2, which was highly conserved among different species and whose 3ʹ-UTR of mRNA contained a complementary site for the seed region of miR-124 (Fig 1A).

A dual luciferase reporter assay was performed to test whether flotillin-2 was a direct target of miR-124 during spermatogenesis. The target region sequence of the flotillin-2 3ʹ-UTR (wt 3ʹ-UTR) and the mutant sequence (mut 3ʹ-UTR) were cloned into a luciferase reporter vector (Fig 1B). These constructed reporter vectors were co-transfected with miR-124 mimics or a non-specific miRNA negative control (miR-Ctrl) into GC2-spd cells. The results showed that miR-124 downregulated the luciferase activity of the flotillin-2 3ʹ-UTR construct, whereas luciferase activity was not significantly decreased in the target region of the mutated mut 3ʹ-UTR construct (Fig 1C). These data suggested that the regulation of miR-124 on flotillin-2 depends on the specific seed region sequence. Moreover, miR-Ctrl did not significantly affect the luciferase activity of either the wt or mut 3ʹ-UTR construct.

MiRNA expression in mammals is tissue and timing specific. Reverse transcriptase PCR (RT-PCR) was used to investigate whether miR-124 is expressed in mouse testicular tissue. The results showed faint expression of miR-124 in adult mouse testes (Fig 1D).

### Function of miR-124 in mouse testes during acrosome biogenesis is a negative factor

**Fig 2:**
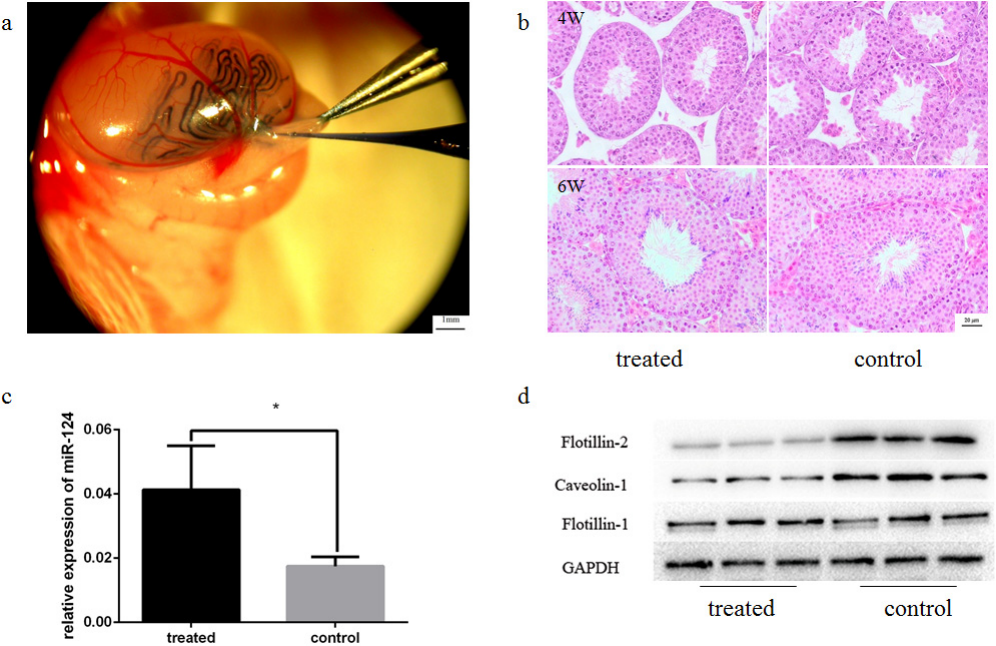
Efficiency of miRNA in vivo. (a) Trypan blue staining was used to trace the seminiferous tubules into which the miRNA was injected; 30–40% of the seminiferous tubules were injected with miRNA. Bar = 1mm. (b) HE staining showed miRNA injection did not lead to seminiferous tubule damage. Bar = 20 μm. (c) qRT-PCR of miR-124 expression. *p< 0.05 compared with control (two-tailed). Error bars indicate SD. (d) Western blot analysis of flotillin-2, caveolin-1, flotillin-1 and GAPDH protein expression. All gels were run under the same conditions.

The relationship between the flotillin-2 protein and miR-124 expression level was analyzed in vivo by intratesticular injection into mouse testes. Round spermatids begin to appear in the testes of 3-week-old mice, and then undergo the first wave of spermiogenesis. Equal amounts of miR-124 mimic or control miRNA were separately injected into each of the paired testes from each mouse in the same manner. The miRNA could only be introduced into 30–40% of the seminiferous tubules to avoid testis tissue damage. To verify the efficiency of miRNA in vivo, the seminiferous tubules were collected from some mice 48 h after injection of miRNA using trypan blue staining (Fig 2A). The results of HE staining of testicular tissue showed that, miRNA injection did not lead to seminiferous tubule damage (Fig 2B). Quantitative real-time PCR (qRT-PCR) was then performed to further evaluate the effects of the injected miRNA. The results demonstrate that miR-124 expression was significantly higher in the miR-124 injected group compared to the negative control group (Fig 2C). Western blotting of seminiferous tubule lysate demonstrated that the expression of flotillin-2 and caveolin-1 protein were consistently suppressed 48h after injection of miR-124, while the expression level of flotillin-1 protein was no significantly different between the two groups (Fig 2D).

**Fig 3:**
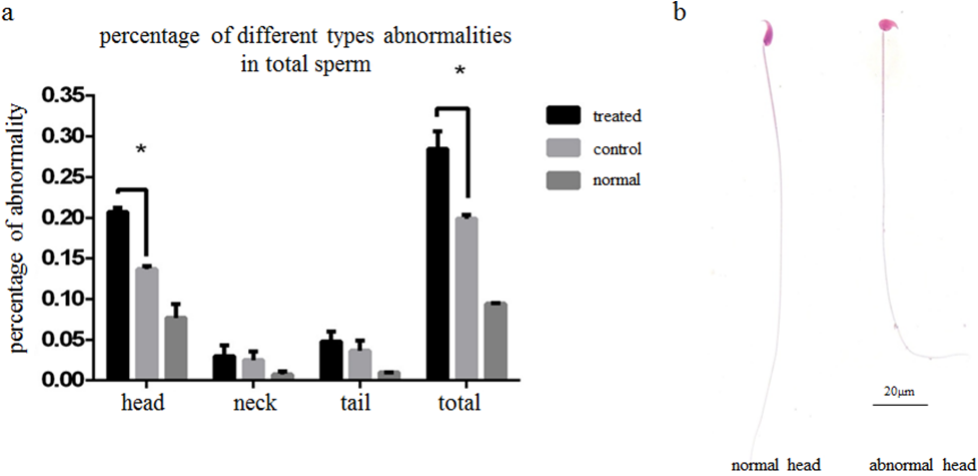
Ordinary optical microscopy of sperm from the caudal epididymis. Paired testes from the same 3 week-old mouse were injected with miR-124 mimic or negative control miRNA, respectively. Different types of sperm abnormality in the cauda epididymis were observed 3 weeks later by ordinary optical microscopy. (a) The ratios were subjected to arcsine square root transformation prior to Student’s t test (two-tailed) * *p*< 0.05 compared with control. Error bars indicate SD. (b) Normal and abnormal head shapes of sperm from the cauda epididymis. Bar = 20 μm.

Sperm were collected from the caudal epididymis for analysis 3 weeks after miR-124 injection. Approximately 19.9% of sperm were abnormal in the control group; by contrast, nearly 28.5% of sperm were abnormal in the miR-124 injected group (*p* = 4.68*10^−5^ vs. control). A large number of sperm from the caudal epididymis of mice treated with miR-124 mimic had abnormal head morphology. However, these abnormalities were rarely observed in sperm in the control group (Fig 3). The proportion of sperm head abnormalities increased markedly to 20.7% in the miR-124 mimic group, which was significantly higher than that of the control group (13.7%, *p* = 0.025).

**Fig 4:**
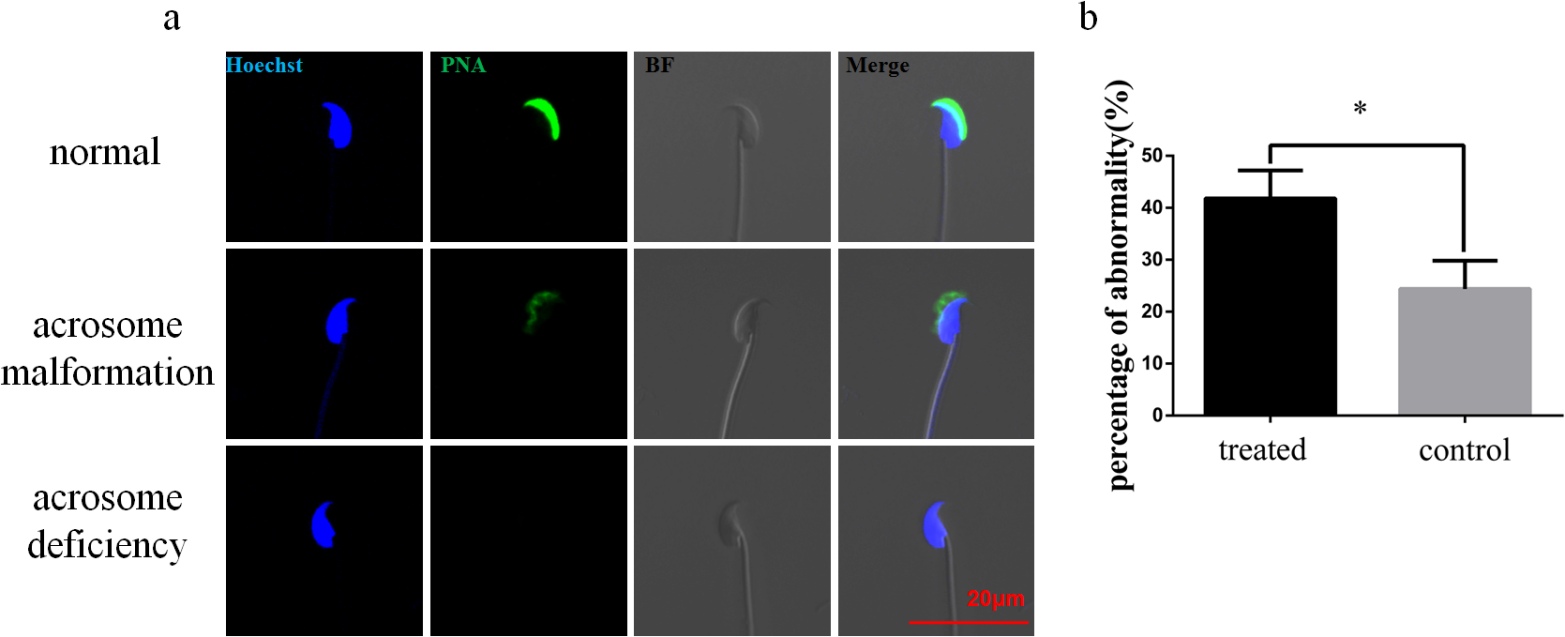
Immunofluorescence detection of the acrosome. (a) Immunofluorescence detection of the acrosome with PNA (green) was performed to illustrate and quantify the sperm acrosome abnormalities. Hoechst was used to nuclei staining (blue); bar = 20μm. (b) The rate of abnormalities including acrosome malformation and deficiency was significantly higher in miR-124 mimic group (41.8%), compared with the control group (24.4%, *p* = 0.021).

Immunofluorescence detection of the acrosome with agglutinin was performed to illustrate and quantify the sperm acrosome abnormalities (Fig 4). The results showed that the abnormality rate, including acrosome malformation and deficiencies was significantly higher in the miR-124 mimic group (41.8%), compared with the control group (24.4%, *p* = 0.021).

**Fig 5:**
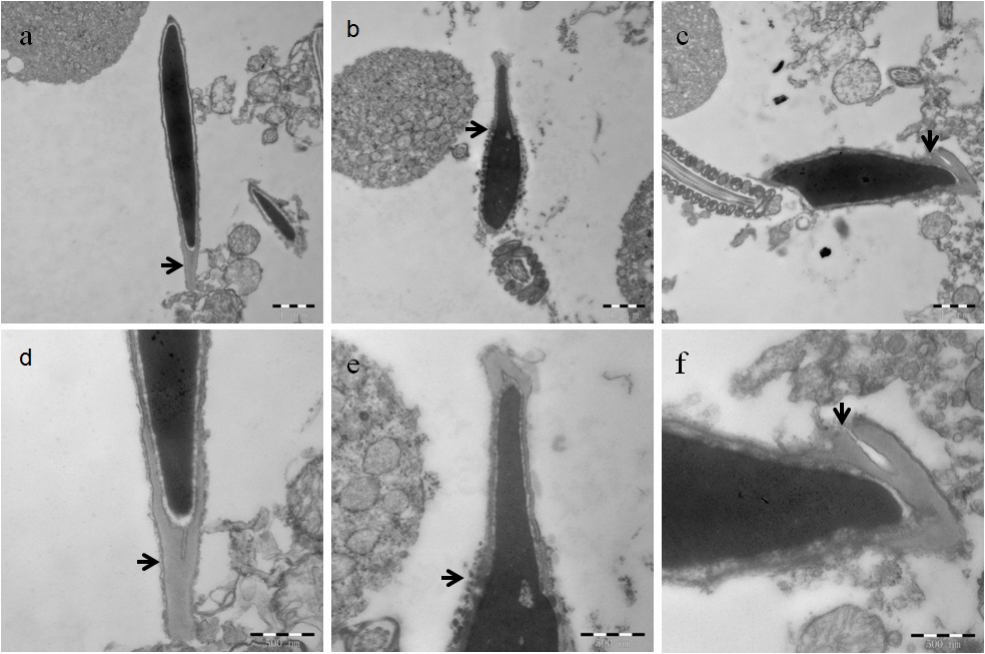
Transmission electron microscopy of sperm from the caudal epididymis. Sperm from the caudal epididymis were analyzed by transmission electron microscopy 3 weeks after miRNA injection. Normal sperm have an elongated condensed head (a, d). The caudal epididymis that corresponding to the testes treated with miR-124 mimic, sperm with acrosome malformations (b, e), or significant acrosome deficiencies (c, f) were observed. Abnormal acrosomes were shown with discrete unfused small acrosomal vesicles around the nucleus; bar = 1μm (a-c), bar = 500nm (d-f).

Transmission electron microscopy was performed 3 weeks after miRNA injection. In the caudal epididymis, normal sperm has an elongated condensed nucleus and one intact acrosome covering the nucleus. However, in the caudal epididymis that corresponded to the testes treated with miR-124 mimic, sperm with acrosome malformation, or a large number of acrosomal deficiencies were observed (Fig 5). These findings showed acrosome abnormalities with discrete unfused small acrosomal vesicles around the nucleus.

## Discussion

In mammals, male gametes are produced in the testes by spermatogenesis, which is a complex process of terminal differentiation by which mature sperms are generated. During spermatogenesis post-transcriptional regulation is particularly essential due to the fact that, germ cells are periodically transcriptionally silenced[28].

Biogenesis of the acrosome, which is a Golgi-derived secretory granule, is a remarkable biological event during spermatogenesis. Clinical studies have confirmed that there is a distinct group of men whose infertility is connected with an abnormal acrosome[29]. The acrosome plays an essential role at the site of sperm–zona (egg) binding during the fertilization process[30]. Defective acrosome biogenesis also leads to sperm DNA compaction failure and consequent defective embryo development[31].

In recent years, miRNAs were identified in mammals[32]. MiRNAs are highly conserved between animals and humans, and it has been estimated that they may regulate 30% of all genes in the human genome[33]. MiRNAs function mainly post-transcriptionally by affecting the stability or translation of their target mRNAs[34–36].

Research has gradually unveiled that a number of novel miRNA molecules are required during spermatogenesis, and in fact some pivotal steps of spermatogenesis rely on a single miRNA molecule. The essential role of miRNAs in the progression of mammalian spermatogenesis and male fertility has been reported [37,38]. For example, it was identified that two genes are vital in spermatogenesis (tgif2 and notch2) as direct targets of miR-34c[39]. Tgif2 inhibits the second meiotic division in spermatogenesis[40]. On the contrary, Notch signaling facilitates germ cell differentiation from spermatogonia[41]. Furthermore, miR-18a directly targets HSF2 (heat shock factor 2), a transcription factor that controls the expression of many genes required for successful spermatogenesis[42]. However, studies investigating the acrosome formation involving miRNAs are not well described.

In our previous study, flotillin-2 was found to play an important role during spermatogenesis, in a similar manner to GOPC and Hrb, which are Golgi-related protein involved in sperm acrosome biogenesis[8]. There is no report on the sperm defects of Flotillin-2 KO mice. Testis-specific conditional knockout mice would be ideal model for the further research. In the current study, bioinformatics analysis was used to predict that miR-124 was a direct and functional target of flotillin-2 via binding to its 3ʹ-UTR. All these observations led to the conclusion that miR-124 could potentially play an important role in flotillin-2 expression, especially in sperm acrosome biogenesis.

A dual luciferase reporter assay system was performed in order to identify the effects of miR-124 on Flotillin-2 expression. MiR-124 was found to be a negative factor which downregulated the expression of flotillin-2. MiR-124 was delivered to the testes of three-week-old male mice by intratesticular injection and analysis via qRT-PCR and western blot confirmed that miR-124 downregulated the expression of flotillin-2. Sperm morphology was analyzed by ordinary optical microscopy and transmission electron microscopy three weeks later. Sperm with obvious deformed heads in the miR-124 mimic group demonstrated acrosome abnormalities. In addition, the expression of caveolin-1 protein was suppressed 48 h after injection of miR-124 and the expression of flotillin-1 protein had no significant difference between the two groups. Although miR-124 has been reported to downregulate flotillin-1 expression in breast cancer[43], this phenomenon was not observed in the current study.

Flotillin-2 was thought to play a direct role in caveolin-independent vesicle trafficking with flotillin-1[44]. Caveolin-1 exists in vesicles in the cytoplasm that can undergo directed movement in the Golgi complex[45,46], and a recent study also found that caveolin-1 might be involved in acrosomal biogenesis[10]. The results from this study indicate that miR-124 might play a role in caveolin-independent vesicle trafficking through downregulating flotillin-2. However, the mechanism of caveolin-1 regulation of acrosomal biogenesis requires elucidation in future work.

This study has revealed, for the first time, that miR-124 modulates the expression of flotillin-2 during spermatogenesis. As previously described, many novel miRNA molecules are required for spermatogenesis, and in fact some pivotal steps of spermatogenesis rely on a single miRNA molecule (e.g. miR-146, miR-221, miR-222 and miR-383). However, spermatogenesis is a complex differentiation process that is prone to error. In addition, alarming adverse trends in semen quality have been reported and the incidence of testicular germ cell tumors and congenital abnormalities of reproductive organs in Western countries is increasing[47].

Thus, these underlying roles for miRNAs in germ cell development have implications both in normal and disease states such as infertility and germ cell tumors in men. Due to the fact that miRNAs have been identified in mature spermatozoa and seminal plasma and their expression profiles appear to be altered in patients with spermatogenic problems, they may emerge as potential biomarkers for diagnosis and classification. Furthermore, by altering targeted miRNAs with complementary anti-miR for loss or gain of a specific function, unique opportunities could potentially be provided for the development of new and more effective therapeutic methods and potent diagnostic tools in the investigation and diagnostics of male infertility.

## Supporting Information

S1 ARRIVE Checklist ARRIVE Guidelines Checklist. (PDF)

S1 Fig Sequence of flotillin-2, reporter vector pLUC-m-Flot2 and reporter vector pLUC-m-Flot2-Mut. According to the algorithms of TargetScan 5.1, the flotillin-2 gene was predicted to be a potential direct target of miR-124. Letters with a green background indicate the sequence of the primer, while letters with a yellow background indicate the microRNA active sites.
(PDF)

S2 Fig Sequence of the mimic control. The mimic control was used both in Luciferase assays and testicular injection.
(PDF)

S3 Fig Western blot analysis. All gels were run under the same conditions. The expression of GAPDH was used as a loading control.
(PDF)

S4 Fig More electron microscopy pictures showed the sperm defects. More electron microscopy pictures showed the abnormal acrosomes, bar = 1μm (a-e), bar = 500nm (f-j).
(PDF)
