# Supplementary material for: Expression of Flotilin-2 and Acrosome Biogenesis Are Regulated by MiR-124 during Spermatogenesis
Source: PLoS One. 2015 Aug 27;10(8):e0136671. doi: 10.1371/journal.pone.0136671 (PMC4551675; doi:10.1371/journal.pone.0136671)
Supplement: S1 Fig — According to the algorithms of TargetScan 5.1, the flotillin-2 gene was predicted to be a potential direct target of miR-124. Letters with a green background indicate the sequence of the primer, while letters with a yellow background indicate the microRNA active sites. (PDF) [file pone.0136671.s002.pdf]

Supplementary information 1 Primer sets and target sequence of plasmid construction

Flot2 3'UTR

GGCTCCTGCACACTCAGCCTCCAGCAGCTGCCTGCCCTTCCAGCGCCTGTTTTAACGTCACAGGGACAGC
GAGAACATTTCTGACTCTGGTGCCTTATTTTGTAGGGACCAGAAGTGCTGCGTGTTCAGGCCATCTCTG
GCTATCTTCCTTTCTCTCCTGTCTGCCTTCCTCTCTCCCATGCCCTCACATTAATGGCCACCTTCATCCA
GCCTGTCTCCTCATCTGCCATATGTGCCTCTTCCTCTGGCTATCTGTTTCTCTCCCCTTTCTCCCCAACT
TCACACACATCATCTACTTTAACTGAAGTTGAAGTTGTGTATGATAATCACTGTCTGTCTGTGAGGAGT
GGCCTCGCTGCTCCTCAGAATCCTGGTGCCTTCAAGTTCTACATGTATCTGTCTGTCCTCCCTGGCCCTG
GCAGAGCCCAGCATGGGTACAAGGGTCCTAGGTAGGACACCCACTCTCCTCTCTCCTGGTCTGAATCTCC
GAGTGCTGAACGCTGTTGCGGGAAGCCCACAACCCACACGCTCTTGCCCTTTGAGGCCTGAGTGGCCAT
GGCCTATTGGCCCTAGAGTAATGCCCTTGACATGCCGAGGCCCAGGCCTGCTGTCATACCTGCCCCTGTC
CCCTGCACCATCACGGAGGCACAGAAGCAAGGCCTCCTGTCTATAGCAGCTTCCTCAGCTTACTACTGCC
TTAGGAGGCCCTGCTTGTGCTCAGGGACATCCCTTCCCTTGGCTTGTCCTTGATTGGTGGGTGGGTCTT
AGTCTGATCCCATTAAGTGTTTCTGGGGTCAATGTCTAGCTCTGTTGGGGACAAGTAAGGTTTATAGGC
CTTCTTCCTGCTAGTGCATGGAGCTCTCCCAGGAGGCCAGGGCTGTGCCAACCCTAAGAGAATCAAGTG
CTGTAGACTGGCCAAACCTCATTCCCTCAATGCCATTTTCTTGGCCAGAATAATGGGGACATGGGAAGC
AGCTAAATATTCTATACCTTTGCACCACTGGTGGCTGAAGAGGCCAATGCCCTGGTGTCCTAGTAGGGG
TACAAGGGTGTCCCGTGGGCCCCGTCCCCACCCCTGGCCCTGCCCAGCCTGTGTAGCTGTTCTGCATGTG
AATGCTGCATGTCTGGTCCGGGATTTGGATGTTGCACTACCCCACTGCCTGTCCCCCTCTGGTGAAAAT
AAAGATCTCTTATACCCAAAAAAAAAAAAAAAAAAA

m-Flot2 3'UTR-F XhoI:cacaactcgagGGCTCCTGCACACTCAGCCT
m-Flot2 3'UTR-R BamHI:aaggatccATAGCCAGAGATGGCCTGAA
m-Flot2
3'UTR-MFA1:tcgagGGCTCCTGCACACTCAGCCTCCAGCAGCTGCCTGCCCTTCCAGCGCCTGTT
m-Flot23'UTR-MRA1:cgttaaAACAGGCGCTGGAAGGGCAGGCAGCTGCTGGAGGCTGAGTGTGCAGGA
GCC
m-Flot2 3'UTR-MFA2:ttaacgTCACAGGGACAGCGAGAACATTTCTGACTCTGCACGGAAATTTTGTA
m-Flot23'UTR-MRA2:ggtcccTACAAAATTTCCGTGCAGAGTCAGAAATGTTCTCGCTGTCCCTGTGA
m-Flot2 3'UTR-MFA3:gggaccAGAAGTGCTGCGTGTTCAGGCCATCTCTGGCTAT
m-Flot23'UTR-MRA3:gatccATAGCCAGAGATGGCCTGAACACGCAGCACTTCT

3'UTR sequencing of Reporter vector pLUC-m-FLot2

GGCTCCTGCACACTCAGCCTCCAGCAGCTGCCTGCCCTTCCAGCGCCTGTTTTAACGTCACAGGGACAGC
GAGAACATTTCTGACTCTGGTGCCTTATTTTGTAGGGACCAGAAGTGCTGCGTGTTCAGGCCATCTCTG
GCTAT

3'UTR sequencing of Reporter vector pLUC-m-FLot2 Mut

GGCTCCTGCACACTCAGCCTCCAGCAGCTGCCTGCCCTTCCAGCGCCTGTTTTAACGTCACAGGGACAGC
GAGAACATTTCTGACTCTGCACGGAAATTTTGTAGGGACCAGAAGTGCTGCGTGTTCAGGCCATCTCTG
GCTAT
