# Supplementary material for: Expression of Flotilin-2 and Acrosome Biogenesis Are Regulated by MiR-124 during Spermatogenesis
Source: PLoS One. 2015 Aug 27;10(8):e0136671. doi: 10.1371/journal.pone.0136671 (PMC4551675; doi:10.1371/journal.pone.0136671)
Supplement: S2 Fig — The mimic control was used both in Luciferase assays and testicular injection. (PDF) [file pone.0136671.s003.pdf]

The sequence of mimic negative control

Sense   5’-UUCUCCGAACGUGUCACGUTT -3’
antisense   5’-ACGUGACACGUUCGGAGAATT-3’
