# Supplementary material for: Expression of Flotilin-2 and Acrosome Biogenesis Are Regulated by MiR-124 during Spermatogenesis
Source: PLoS One. 2015 Aug 27;10(8):e0136671. doi: 10.1371/journal.pone.0136671 (PMC4551675; doi:10.1371/journal.pone.0136671)

Supplementary information 3 Western blot analysis

Blots of flotillin-2

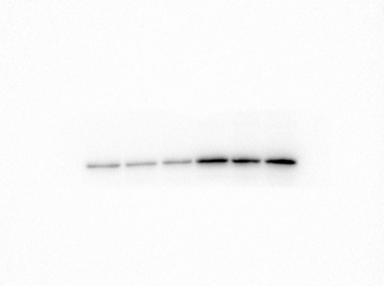

Blots of flotillin-1

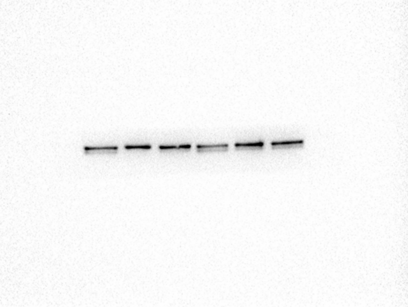

Blots of caveolin-2

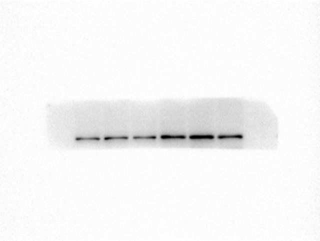

Blots of gapdh

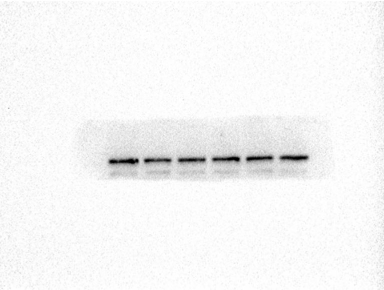

Supplement: S3 Fig — All gels were run under the same conditions. The expression of GAPDH was used as a loading control. (PDF) [file pone.0136671.s004.pdf]
