# Supplementary material for: Expression of Flotilin-2 and Acrosome Biogenesis Are Regulated by MiR-124 during Spermatogenesis
Source: PLoS One. 2015 Aug 27;10(8):e0136671. doi: 10.1371/journal.pone.0136671 (PMC4551675; doi:10.1371/journal.pone.0136671)

Supplementary information 4 More electron microscopy pictures showed the sperm defects.

More electron microscopy pictures showed the abnormal acrosomes bar=1μm (a-e) , bar=500nm (f-j).

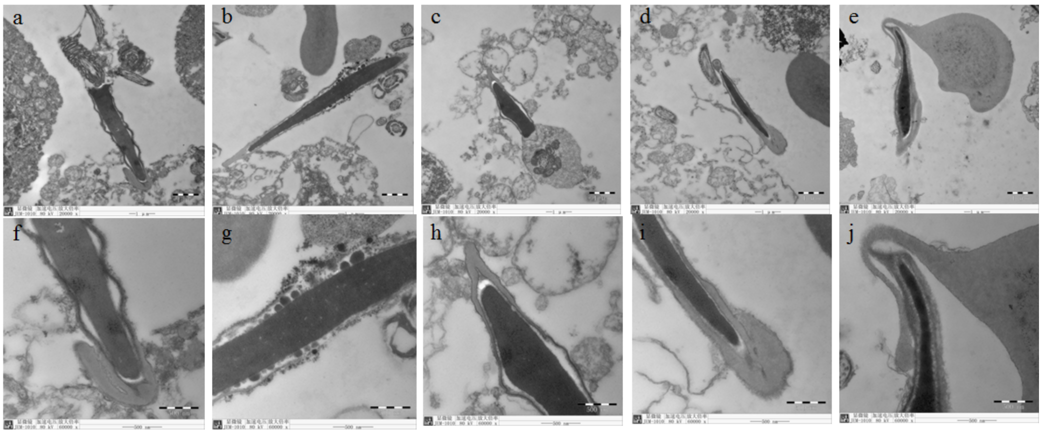

Supplement: S4 Fig — More electron microscopy pictures showed the abnormal acrosomes, bar = 1μm (a-e), bar = 500nm (f-j). (PDF) [file pone.0136671.s005.pdf]
